# Impact of Altered Mineral Metabolism on Pathological Cardiac Remodeling in Elevated Fibroblast Growth Factor 23

**DOI:** 10.3389/fendo.2018.00333

**Published:** 2018-06-21

**Authors:** Maren Leifheit-Nestler, Beatrice Richter, Melis Basaran, Julia Nespor, Isabel Vogt, Ioana Alesutan, Jakob Voelkl, Florian Lang, Joerg Heineke, Stefanie Krick, Dieter Haffner

**Affiliations:** ^1^Department of Pediatric Kidney, Liver and Metabolic Diseases, Pediatric Research Center, Hannover Medical School, Hannover, Germany; ^2^Division of Nephrology, Department of Medicine, University of Alabama at Birmingham, Birmingham, AL, United States; ^3^Department of Internal Medicine and Cardiology, Center for Cardiovascular Research, Charité University Medicine, Berlin, Germany; ^4^Berlin Institute of Health (BIH), Berlin, Germany; ^5^Department of Physiology I, University of Tuebingen, Tuebingen, Germany; ^6^Department of Cardiology and Angiology, Experimental Cardiology, Rebirth-Cluster of Excellence, Hannover Medical School, Hannover, Germany; ^7^Division of Pulmonary, Allergy and Critical Care Medicine, University of Alabama at Birmingham, Birmingham, AL, United States

**Keywords:** fibroblast growth factor 23, klotho deficiency, mineral metabolism, left ventricular hypertrophy, cardiac fibrosis, klotho hypomorphic mice, *Hyp* mice

## Abstract

Clinical and experimental studies indicate a possible link between high serum levels of fibroblast growth factor 23 (FGF23), phosphate, and parathyroid hormone (PTH), deficiency of active vitamin D (1,25D) and klotho with the development of pathological cardiac remodeling, i.e., left ventricular hypertrophy and myocardial fibrosis, but a causal link has not been established so far. Here, we investigated the cardiac phenotype in klotho hypomorphic (*kl/kl*) mice and *Hyp* mice, two mouse models of elevated FGF23 levels and klotho deficiency, but differing in parameters of mineral metabolism, by using histology, quantitative real-time PCR, immunoblot analysis, and serum and urine biochemistry. Additionally, the specific impact of calcium, phosphate, PTH, and 1,25D on hypertrophic growth of isolated neonatal rat cardiac myocytes was investigated *in vitro*. *Kl/kl* mice displayed high serum Fgf23 levels, increased relative heart weight, enhanced cross-sectional area of individual cardiac myocytes, activated cardiac Fgf23/Fgf receptor (Fgfr) 4/calcineurin/nuclear factor of activated T cell (NFAT) signaling, and induction of pro-hypertrophic NFAT target genes including *Rcan1, bMHC*, brain natriuretic peptide (*BNP*), and atrial natriuretic peptide (*ANP*) as compared to corresponding wild-type (WT) mice. Investigation of fibrosis-related molecules characteristic for pathological cardiac remodeling processes demonstrated ERK1/2 activation and enhanced expression of Tgf-β1, *collagen I*, and Mmp2 in *kl/kl* mice than in WT mice. In contrast, despite significantly elevation of serum and cardiac Fgf23, and reduced renal *klotho* expression, *Hyp* mice showed no signs of pathological cardiac remodeling. *Kl/kl* mice showed enhanced serum calcium and phosphate levels, while *Hyp* mice showed unchanged serum calcium levels, lower serum phosphate, and elevated serum iPTH concentrations compared to corresponding WT mice. In cultured cardiac myocytes, treatment with both calcium or phosphate significantly upregulated endogenous *Fgf23* mRNA expression and stimulated hypertrophic cell growth and expression of pro-hypertrophic genes. The treatment with PTH induced hypertrophic cell growth only, and stimulation with 1,25D had no significant effects. In conclusion, our data indicate that *Hyp* mice, in contrast to *kl/kl* mice appear to be protected from pathological cardiac remodeling during conditions of high FGF23 levels and klotho deficiency, which may be due, at least in part, to differences in mineral metabolism alterations, i.e., hypophosphatemia and lack of hypercalcemia.

## Introduction

Patients with chronic kidney disease (CKD) (1–3) and patients with heart failure (4–8) display abnormalities in mineral metabolism, which are associated with cardiovascular diseases (CVD). The phosphaturic hormone fibroblast growth factor (FGF) 23 is synthesized and secreted by osteocytes in response to various stimuli (9) to increase renal phosphate excretion by inhibiting the expression of sodium–phosphate co-transporters NaPi-2a and NaPi-2c (10, 11). Moreover, FGF23 suppresses parathyroid hormone (PTH) expression and secretion (12, 13) and inhibits renal formation of 1,25(OH)_2_D_3_ (1,25D, calcitriol) by downregulating 1α-hydroxylase (Cyp27b1) and upregulating 24-hydroxylase (Cyp24a1) (11, 14, 15). Klotho is an endocrine hormone primarily produced in the kidney (16) and functions as co-receptor for FGF23, whereby it increases the binding affinity of FGF23 to its FGF receptors (FGFRs) and, therefore, mediates different signaling pathways including renal phosphate and 1,25D metabolism (17–19).

With declining kidney function, the endocrine network of mineral metabolism becomes altered, which leads to elevated serum levels of FGF23, phosphate, and PTH in addition to a deficiency of 1,25D and klotho (20). All these parameter represent key risk factors for the development of endothelial dysfunction, left ventricular hypertrophy (LVH), myocardial fibrosis, and contribute to the overall cardiovascular mortality in CKD and non-CKD patients (1, 6, 21–26). We previously showed that FGF23 directly targets the heart and promotes LVH by binding to FGFR4 on cardiac myocytes activating phospholipase C gamma (PLCγ)/calcineurin/nuclear factor of activated T cells (NFAT) to induce pro-hypertrophic gene expression independently of its co-receptor klotho that is not expressed in the heart (16, 27–29). Moreover, we demonstrated that FGF23 contributes to pathologic cardiac remodeling and promotes the pro-fibrotic crosstalk between cardiac myocytes and fibroblasts resulting in enhanced cardiac hypertrophy and fibrosis in the absence of klotho (30).

Besides high FGF23 levels, klotho deficiency also seems to be associated with cardiac dysfunction in humans and rodents (25, 29, 31), and rescuing the availability of klotho by genetic overexpression or intravenous delivery of soluble klotho shows beneficial outcomes including amelioration of cardiac hypertrophy in klotho-deficient uremic mice and suppression of cardiac fibroblast activation and collagen synthesis (31–33). In addition, klotho ameliorates FGF23-mediated oxidative stress by inducing nitric oxide synthesis and degrading reactive oxygen species in endothelial cells (34). Furthermore, klotho protects against myocardial hypertrophy by reducing indoxyl sulfate-mediated oxidative stress *in vitro* and *in vivo* (35).

To date, the impact of high phosphate levels for the development of cardiomyopathy in the setting of high FGF23 levels is controversially discussed. Increased serum phosphate is associated with cardiovascular mortality in CKD (25, 36) and FGF23 mediates renal phosphate excretion only in the presence of klotho (19). Normalization of serum phosphate and FGF23 levels by dietary phosphate restriction in 5/6 nephrectomized klotho-deficient mice did not abrogate the development of cardiac hypertrophy suggesting that reduced klotho contributes to uremic cardiomyopathy independent of phosphate and FGF23 (32). In contrast, cardiac hypertrophy and fibrosis correlated with high phosphate levels in uremic and non-uremic klotho-deficient mice, and genetically induced elevation of soluble klotho ameliorated phosphate-induced hypertrophic growth of cardiac myocytes *in vivo* (31). The association between FGF23 and cardiac remodeling in klotho-deficient animals suggests that FGF23 affects cardiac hypertrophy and fibrosis only in states of high phosphate and klotho deficiency. Thus, the interplay between increased FGF23, phosphate, and reduced klotho participates in the development of CVD. However, the direct impact of each single factor on pathologic cardiac remodeling is still not clear. Here, we test whether elevated FGF23 levels in klotho deficiency invariably leads to cardiac hypertrophy and fibrosis and whether different alterations in mineral metabolism play a role in these effects. Therefore, we compared two mouse models with genetically elevated serum FGF23 levels and reduced renal klotho availability, *Hyp* mice, and klotho hypomorphic (*kl/kl*) mice, with respect to the development of a pathologic cardiac phenotype. The *Hyp* mouse is a murine homolog to the human disease of X-linked dominant hypophosphatemia resembling elevated circulating FGF23 concentrations resulting in renal phosphate wasting, hypophosphatemia, decreased renal 1,25D synthesis, and defects in bone mineralization (37, 38). Klotho hypomorphic mice present with high plasma concentrations of FGF23, 1,25D, and phosphate, resulting in severe soft tissue calcification and premature aging (16). In addition, we used neonatal rat ventricular myocytes (NRVM) to study the role of parameters of altered mineral metabolism including calcium, phosphate, PTH, and 1,25D for the induction of cardiac hypertrophy.

## Materials and Methods

### Animal Experiments

All experimental procedures were approved by the State Office committee on animal welfare Lower Saxony for *Hyp* mice and Baden-Württemberg for *kl/kl* mice and performed in accordance with national animal protection guidelines from Directive 2010/63/EU of the European Parliament on the protection of animals used for scientific purposes.

Hemizygous male B6.Cg-Phex^Hyp^/J (*Hyp*) mice (strain no. 000528; Jackson Laboratory, Bar Harbor, ME, USA) with X-linked semidominant mutation in the *Phex* gene causing defects in phosphate metabolism and male wild-type (WT) littermates produced from breeding of heterozygous females (*Hyp/*+) with C57BL/6J WT males were used in this study. Mice were fed normal rodent chow containing 600 IU/kg cholecalciferol, 0.7% calcium, and 0.5% phosphate (#1324, Altromin, Lage, Germany) *ad lib*. The origin of homozygous klotho hypomorphic mice (*kl/kl*), breeding, and genotyping were described previously (16). Five to 13 mice per group were used in this study and sacrificed at 6–8 weeks of age. Blood was collected *via* cardiac puncture, and hearts were isolated and prepared for histological and biochemical analyses. For serology, blood was centrifuged at 4°C and 13,000 rcf for 20 min. Serum supernatant was collected, stored at −80°C, and subsequently analyzed *via* ELISA techniques for C-term FGF23, intact FGF23, and 1-84 PTH (each from Immutopics, San Clemente, CA, USA), and spectrophotometrically for calcium and phosphate (each from DiaSys Diagnostic Systems GmbH, Holzheim, Germany).

### Isolation of NRVM

Neonatal rat ventricular myocytes (NRVM) were isolated using a standard isolation system (Worthington Biochemical Corporation) (39). In brief, hearts from 1- to 2-days-old Sprague Dawley rats were harvested, minced in calcium- and magnesium-free Hank’s Balanced Salt Solution (HBSS) followed by tissue digestion with 50 µg/mL trypsin at 4°C for 20–24 h. Soybean trypsin inhibitor in HBSS was added and the tissue was further digested with collagenase (in Leibovitz L-15 medium) under slow rotation (15 rpm) at 37°C for 45 min. Cells were homogenized and resuspended 20 times with a standard 10 mL serological pipette and filtered twice through a 70-µm cell strainer (BD Falcon). After incubation at room temperature for 20 min, cells were centrifuged at 100 × *g* for 5 min and cell pellet was re-suspended in plating medium Dulbecco’s Modified Eagle Medium (DMEM) with 20% M199 (Invitrogen), 15% fetal bovine serum (FBS; Invitrogen), and 1% penicillin/streptomycin solution (P/S; Invitrogen).

Cells were plated on glass and plastic surfaces pre-coated with laminin (Invitrogen; 10 µg/mL in PBS) at room temperature for 1 h. For immunofluorescence analysis, 3 × 10^5^ cells were seeded per well on pre-coated glass coverslips in 24-well plates and for RNA isolations, 8 × 10^5^ cells were seeded in 6 cm-culture dishes. Cells were left in plating medium at 37°C over night. After starvation in maintenance medium DMEM with 20% M199, 1% insulin-transferrin-sodium selenite solution (ITS; Sigma-Aldrich), and 1% P/S, isolated NRVM were stimulated in duplicates in maintenance medium in the presence of 3 mM calcium, 1 mM phosphate, 10 nM PTH, 10 nM calcitriol, or vehicle, respectively, for 48 h. At least six independent cell isolations were used for all experiments.

### Histological Analysis

Formalin-fixed paraffin-embedded heart tissue samples were deparaffinized in xylene, hydrated through a series of graded alcohols. For the quantification of cardiac myocyte size, fixed cardiac mid-chamber (MC) (33) sections were incubated with wheat germ agglutinin (WGA) Alexa Fluor555 (Invitrogen) at 5 µg/mL in PBS for 1 h to visualize cellular borders of individual cardiac myocytes. 4′,6-diamidino-2-phenylindole (DAPI; 0.2 µg/mL) was used for nuclear staining in the dark for 15 min. Representative immunofluorescence images of cardiac tissue were taken on a Zeiss AxioObserver Z1 microscope (Carl Zeiss) with a Plan-Apo 63×/N.A. 1.4 oil objective. ZEN Software (Carl Zeiss) was used to measure myocardial cross-sectional area in square micrometer of 100 cardiac myocytes in average.

For the detection of myocardial fibrosis and visualization of fibrillar collagen fibers, MC sections were stained with picrosirius red (Sigma-Aldrich) as described previously (28). In brief, deparaffinized MC sections were incubated with picrosirius red for 60 min followed by mounting in non-aqueous mounting medium and analyzed by bright field and polarized microcopy using a Keyence BZ-9000^®^ microscope with a 20× objective. In addition, for polarized light microscopy, two polarization filters were used in a rectangular orientation, positioned above and below the sample.

### Immunocytochemistry and Morphometry of NRVM

To analyze hypertrophic growth of isolated NRVM on laminin-coated glass coverslips after 48 h of treatment with calcium, phosphate, PTH, and 1,25D, NRVM were fixed in 2% PFA in 5 mg/mL sucrose for 5 min and permeabilized in 1% Triton X-100 in PBS for 10 min followed by incubation with mouse monoclonal antibody against sarcomeric α-actinin (1:1,000 dilution; EA-53; Sigma-Aldrich). Cy3-conjugated goat-anti mouse (Jackson Immuno Research) was used as secondary antibody at 1:300 dilution. To visualize nuclei, fixed cells were incubated with DAPI (400 ng/mL in PBS) for 10 min. Immunofluorescence images were taken on a Zeiss AxioObserver Z1 microscope (Carl Zeiss) with a 40× objective. Myocyte cross-sectional area was measured based on α-actinin-positive staining using Carl Zeiss ZEN software. At least 100 cells per stimulation were quantified for the determination of cardiac myocyte cross-sectional area.

### RNA Isolation and Quantitative Real-Time PCR (qRT-PCR) Analysis

For RNA isolation of snap-frozen mouse myocardial tissue or NRVM, RNeasy Mini Kit (Qiagen) was used according to the manufacturer protocol. Total RNA (500 ng) was transcribed into cDNA using QuantiTect Reverse Transcription Kit (Qiagen) and qRT-PCR was performed in triplicates (20 ng cDNA per reaction) with appropriate primers in 5′–3′orientation (Table 1) using QuantiFAST SYBR Green PCR Kit including ROX dye (Qiagen). Forty-five cycles (95°C, 10 s; 60°C, 30 s) were performed on an ABI prism 7900HT Fast system (Applied Biosystems). Relative gene expression values, adjusted for the same CT-threshold and baseline settings, were calculated according to the 2^−ΔΔCT^ method (40) using *Gapdh* as housekeeping gene (SDS Software v2.3, Applied Biosystems).

**Table 1:** The following oligonucleotides shown in 5′–3′orientation were used as primers for quantitative RT-PCR analyses.

Gene	Orientation	Primer sequence (5′–3′)
Mouse Fgf23		Mm_Fgf23_1_SG QuantiTect Primer Assay QT01066772 (Qiagen, Hilden, Germany)

Mouse Fgfr1	Forward	TGC CAG CTG CCA AGA CGG TG
	Reverse	AAG GAT GGG CCG GTG AGG GG

Mouse Fgfr4	Forward	GGC TAT GCT GTG GCC GCA CT
	Reverse	GGT CTG AGG GCA CCA CGC TC

Mouse Rcan1	Forward	CCC GTG AAA AAG CAG AAT GC
	Reverse	TCC TTG TCA TAT GTT CTG AAG AGG G

Mouse aMHC	Forward	ACT GTG GTG CCT CGT TCC
	Reverse	GCC TCT AGG CGT TCC TTC TC

Mouse bMHC	Forward	AGG CAA GGC AAA GAA AGG CTC ATC
	Reverse	GCG TGG AGC GCA AGT TTG TCA TAA

Mouse ANP	Forward	ATT GAC AGG ATT GGA GCC CAG AGT
	Reverse	GA CAC ACC ACA AGG GCT TAG GAT

Mouse BNP	Forward	CCA GAT GAT TCT GCT CCT GC
	Reverse	TGA ACT ATG TGC CAT CTT GG

Mouse Col1	Forward	CCG CTG GTC AAG ATG GTC
	Reverse	CCT CGC TCT CCA GCC TTT

Mouse Mmp2	Forward	AAC TAC GAT GAT GAC CGG AAG TG
	Reverse	TGG CAT GGC CGA ACT CA

Mouse Tgfb1	Forward	TTG CTT CAG CTC CAC AGA GA
	Reverse	TGG TTG TAG AGG GCA AGG AC

Mouse Klotho	Forward	TGG GAA GGT TTT GTC CAG AAG A
	Reverse	AGA AAC GAG ATG AAG ACC AGC A

Mouse Gapdh	Forward	TAT GTC GTG GAG TCT ACT GG
	Reverse	AGT GAT GGC ATG GAC TGT GG

Rat Fgf23	Forward	GCA ACA TTT TTG GAT CGT ATC A
	Reverse	GAT GCT TCG GTG ACA GGT AGA

Rat Rcan1	Forward	CTC ACA CAC GTG GAC CAC CA
	Reverse	CGC CCA ATC CAG ACA AAC AG

Rat bMHC	Forward	CTC CAG AAG AGA AGA ACT CC
	Reverse	CCA CCT GCT GGA CAT TCT GC

Rat ANP	Forward	AAA TCC CGT ATA CAG TGC GG
	Reverse	GGA GGC ATG ACC TCA TCT TC

Rat BNP	Forward	CCA GAA CAA TCC ACG ATG C
	Reverse	TCG AAG TCT CTC CTG GAT CC

Rat Gapdh	Forward	ACT CCA CGA CAT ACT CAG CAC
	Reverse	CAT CAA CGA CCC CTT CAT T

### Protein Isolation and Immunoblotting

For protein extraction from snap-frozen myocardial specimens of the left ventricle of *Hyp* and *kl/kl* mice, 30 mg tissue was homogenized in 250 µL RIPA extraction buffer (50 mM Tris–HCl pH 7.4, 150 mM NaCl, 1% NP-40, 0.25% Na-desoxycholate, 1 mM EDTA) with protease (P8340, Sigma-Aldrich) and phosphatase (S65208, Sigma-Aldrich) inhibitors, sonicated two times and incubated on ice for 30 min. Cell lysates were centrifuged at 13,000 rpm and 4°C for 10 min, protein concentration was quantified using BCA test, 100 µg total protein was boiled in sample buffer and analyzed by SDS-PAGE and immunoblotting. Antibodies to FGF23 (1:1,000; ab98000, Abcam), Calcineurin A (1:10,000; ab52761, Abcam), NFATc4 (1:200; sc-13036, Santa Crus Biotechnology Inc.), Rcan1 (1:1,000; SAB2101967, Sigma), pERK1/2 and ERK1/2 (each 1:1,000; #4370 and #9107, Cell Signaling Technology), connective tissue growth factor (Ctgf) (1:1,000; ab6992, Abcam), and GAPDH (1:1,000; #2118, Cell Signaling Technology) were used as primary antibodies in 5% BSA in LI-COR blocking buffer/TBS (1:2) over night, and IRDye^®^ secondary goat-anti-mouse and goat-anti-rabbit (LI-COR Biosciences) were used as secondary antibodies. Primary antibodies to collagen 1 (1:1,000; ab34710, Abcam), MMP2 (1:500; ab92536, Abcam), and TGF-β1 (1:500; ab179695, Abcam) were used in 5% milk in TBS-T over night followed by using HRP-labeled secondary antibodies and enhanced chemiluminescent substrate. The Odyssey Imager (LI-COR Biosciences) was used for protein detection and quantification.

### Statistical Analysis

Data are presented as mean ± SEM if not indicated otherwise. Comparison between groups of *Hyp* or *kl/kl* mice and its respective WT littermates were done by unpaired *t*-test in case of normally distributed data or Mann–Whitney *U* test in case of non-Gaussian distributions, respectively (GraphPad Prism Software version 6.0). Different groups of NRVM were compared by one-way ANOVA and Bonferroni’s multiple comparison *post hoc* tests. Two-tailed *P* values of <0.05 were considered statistically significant.

## Results

### Circulating Fgf23 Levels and Endogenous Cardiac Fgf23 Synthesis Are Elevated in *Hyp* and *kl/kl* Mice

**Figure 1:**
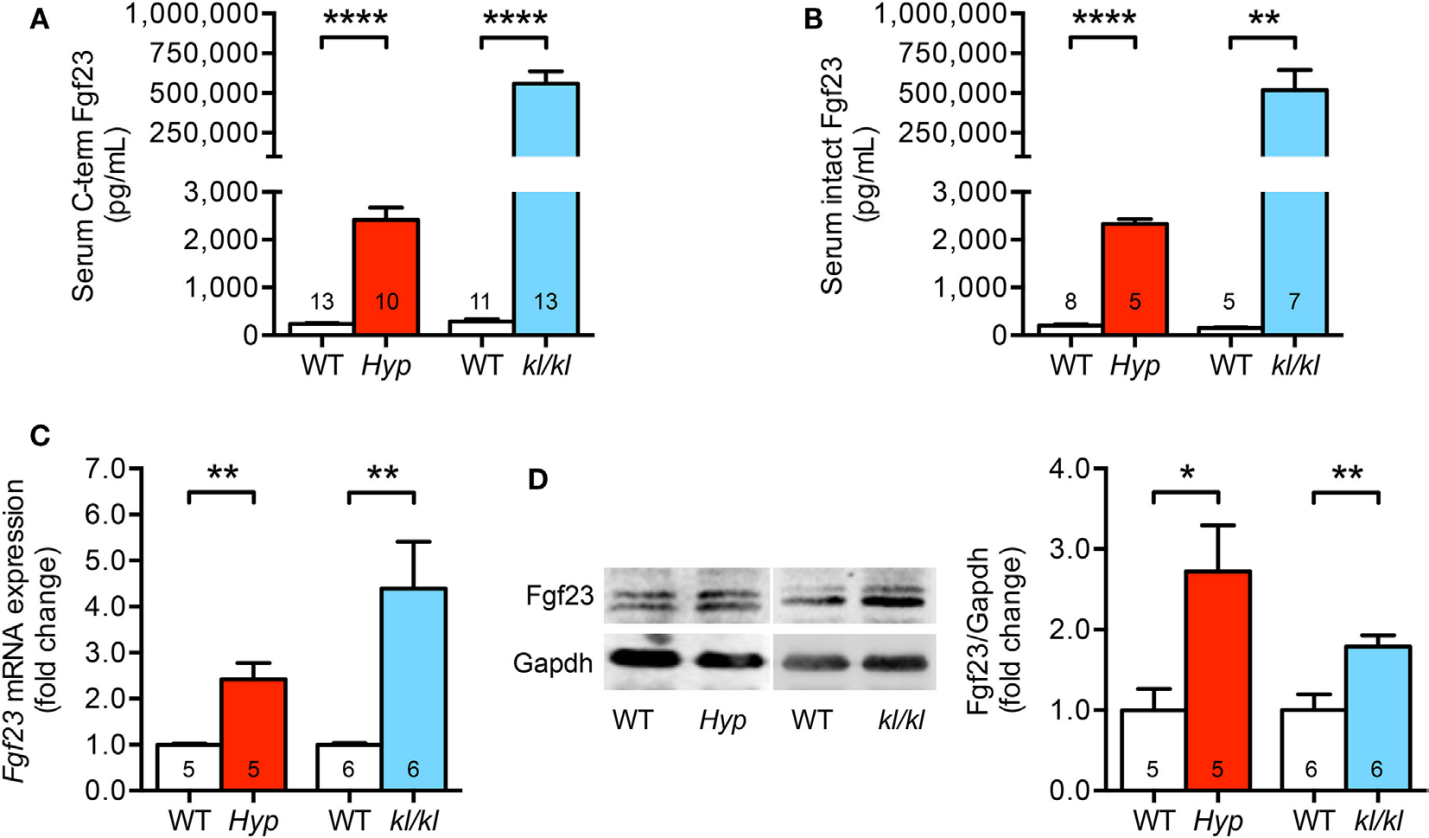
Serum Fgf23 levels and cardiac Fgf23 expression are elevated in *Hyp* and *kl/kl* mice. **(A)** Increased serum C-term Fgf23 levels in both *Hyp* and *kl/kl* mice as compared to respective wild-type mice. **(B)** Elevation of intact Fgf23 concentrations in the serum of *Hyp* and *kl/kl* mice than in respective control mice. **(C)** *Fgf23* mRNA levels are increased in cardiac tissue of both mouse lines demonstrated by quantitative real-time PCR after normalization to *Gapdh*. **(D)** Representative immunoblot and respective quantification show the elevation of Fgf23 protein levels in total heart lysates of *Hyp* and *kl/kl* animals (Gapdh served as loading control). Values are presented as mean ± SEM; numbers in each bar graph represent the *n-*values used for the respective measurement; *P < 0.05, **P < 0.01, ****P < 0.0001.

First, we investigated circulating and cardiac levels of Fgf23 in *Hyp* and *kl/kl* mice. *Hyp* mice presented with 10-fold higher levels of both serum C-term and intact Fgf23 when compared to WT littermates. In *kl/kl* mice, circulating C-term and intact Fgf23 concentrations were 2,000- and 3,300-fold higher compared to respective WT controls (Figures 1A,B). In the heart tissue of *Hyp* and *kl/kl* mice, endogenous cardiac *Fgf23* mRNA expression and full-length biological active Fgf23 protein were significantly upregulated compared to respective WT controls (Figures 1C,D). Thus, both mouse models showed elevated circulating and cardiac Fgf23 levels, although serum Fgf23 concentrations appeared to be higher in *kl/kl* compared to *Hyp* mice.

### Homozygous *kl/kl*, but Not *Hyp* Mice, Develop Cardiac Hypertrophy

**Figure 2:**
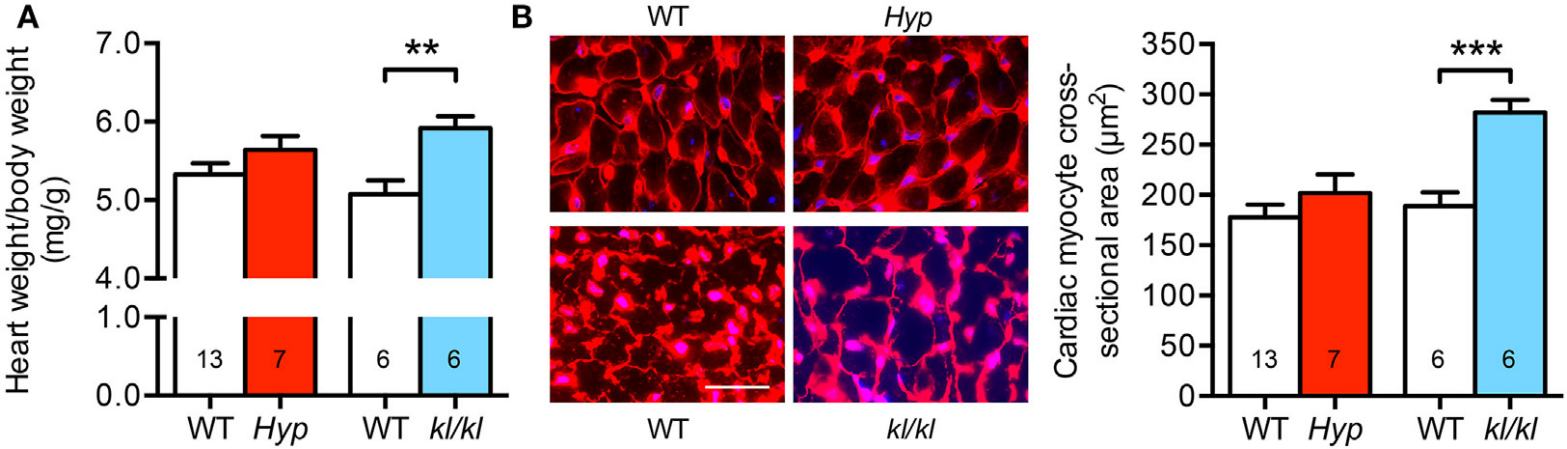
Development of cardiac hypertrophy in *kl/kl* mice. **(A)** The relative heart weight to body weight ratio is increased only in *kl/kl* mice. **(B)** Immunofluorescence co-staining for wheat germ agglutinin (WGA) Alexa Fluor555 (red) and cell nuclei (blue) demonstrates enlarged size of cardiac myocytes in *kl/kl* mice but not in *Hyp* mice (magnification, 63×; scale bar, 25 µm). Values are presented as mean ± SEM; numbers in each bar graph represent the *n-*values used for the respective measurement; **P < 0.01, ***P < 0.001.

Next, we investigated the development of cardiac hypertrophy in both mouse models. Due to growth retardation caused by abnormal mineral metabolism in *Hyp* mice, we calculated the relative heart weight on the basis of heart weight to body weight ratio. When compared to WT controls, the relative heart weight of *kl/kl* mice was 0.84 ± 0.15 mg/g higher (Figure 2A). In addition, *kl/kl* mice presented with 282 ± 13 μm^2^ cross-sectional area of individual cardiac myocytes compared to 189 ± 14 μm^2^ in WT littermates demonstrated by WGA staining of the myocyte cell borders in heart tissue sections. In contrast, *Hyp* mice showed unaltered cardiac myocytes cell size compared to WT littermates (202 ± 19 versus 178 ± 13 μm^2^; *P* = 0.287) (Figure 2B). Thus, it appears that only *kl/kl* mice display the development of cardiac hypertrophy.

### Cardiac-Specific Fgfr4/Calcineurin/Nfat Signaling Is Only Activated in *kl/kl* Mice

**Figure 3:**
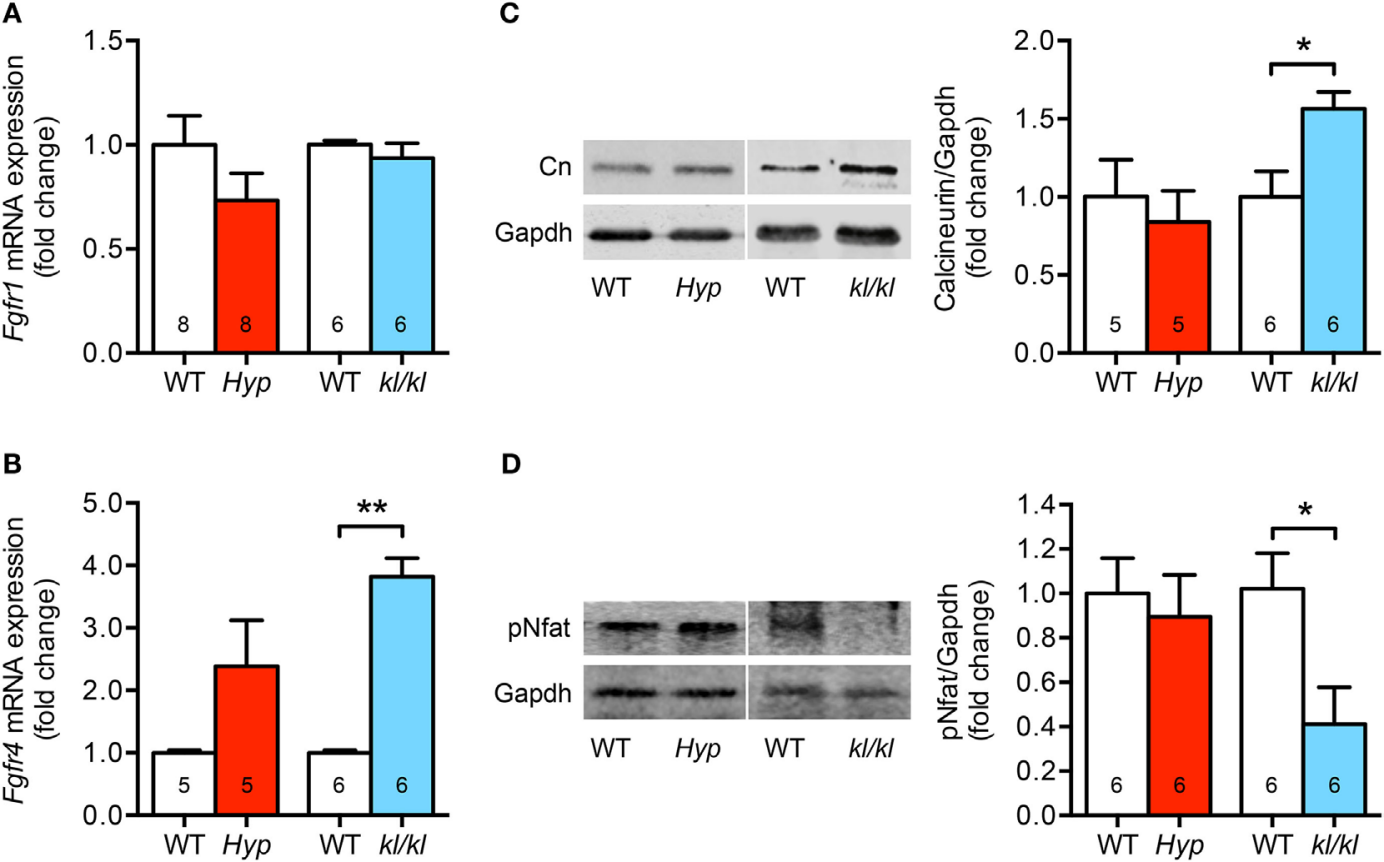
The cardiac Fgfr4/calcineurin/nuclear factor of activated T cell signaling is activated in *kl/kl* mice. **(A)** Quantitative real-time PCR analysis reveals no significant differences in *Fgfr1* mRNA expression in cardiac tissue from both mouse lines compared to their respective wild-type controls. **(B)** *Fgfr4* mRNA expression is elevated in hearts from *kl/kl* mice as well as *Hyp* mice; however, the differences are statistically significant only for the *kl/kl* animals. **(C)** Representative immunoblot and quantification indicate an upregulation of cardiac calcineurin protein expression only for *kl/kl* mice (Gapdh served as loading control). **(D)** Representative immunoblot and quantification of Nfat activation show reduced Nfat phosphorylation in cardiac tissue from *kl/kl* mice. Values are presented as mean ± SEM; numbers in each bar graph represent the *n-*values used for the respective measurement; *P < 0.05, **P < 0.01.

Since Fgfr1 and Fgfr4 are the main Fgfrs expressed in the myocardial tissue of humans and rodents (27, 29), and each induces pathological cardiac remodeling through a different pathway, we next wanted to know whether both receptors were altered in the heart of *Hyp* and *kl/kl* mice. Cardiac *Fgfr1* mRNA levels were unchanged in *kl/kl* mice, but tended to be lower in *Hyp* mice; however, the difference did not reach statistical significance (*P* = 0.184) (Figure 3A). In contrast, cardiac *Fgfr4* mRNA expression was 3.8 ± 0.3-fold upregulated in *kl/kl* mice compared to controls. In *Hyp* mice, a trend of *Fgfr4* elevation was recognized when compared to their respective WT controls (2.4 ± 0.7-fold; *P* = 0.097) (Figure 3B). Calcineurin protein levels, activated by Fgfr4 *via* PLCγ, were 1.6 ± 0.1-fold enhanced in heart tissue lysates from *kl/kl* mice (*P* = 0.017), but not from *Hyp* mice (Figure 3C). Moreover, the transcription factor Nfat was clearly de-phosphorylated (0.41 ± 0.16-fold; *P* = 0.027) and thereby activated in heart tissue only from *kl/kl* mice compared to WT littermates (Figure 3D). Taken together, the pro-hypertrophic Fgfr4/calcineurin/NFAT signaling pathway is induced in *kl/kl*, but not in *Hyp* mice.

### Pro-Hypertrophic NFAT Target Markers Are Upregulated in *kl/kl* but Not in *Hyp* Mice

**Figure 4:**
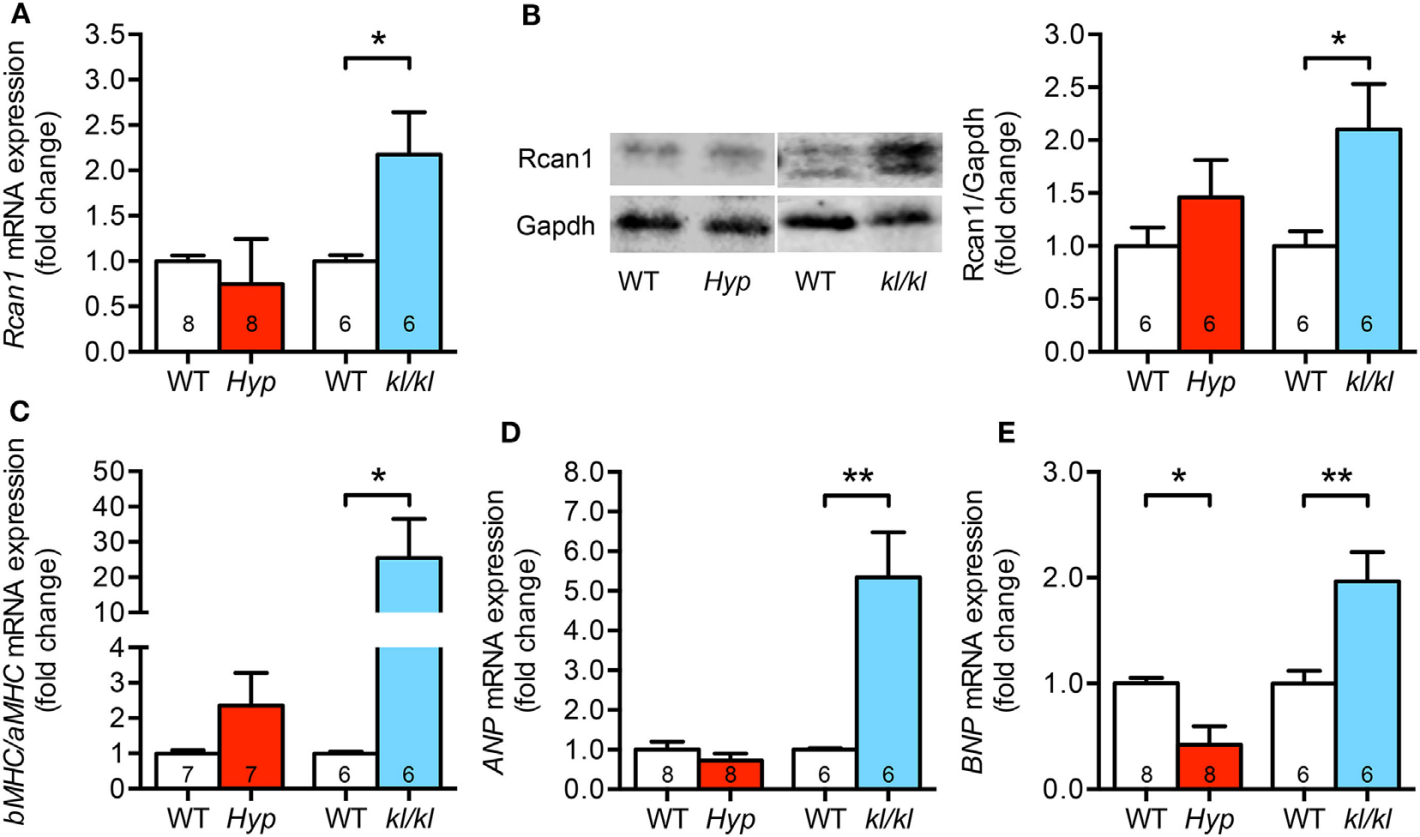
*Kl/kl* mice show an upregulation of cardiac pro-hypertrophic nuclear factor of activated T cell target genes. **(A,B)** Rcan1 expression is increased only in cardiac tissue from *kl/kl* mice demonstrated by quantification of mRNA levels by real-time PCR and protein levels by western blotting. **(C)** Quantification of *bMHC* and *aMHC* mRNA expression shows the induction of fetal gene program in heart tissue from *kl/kl* mice but not from *Hyp* mice. **(D)** *ANP* mRNA expression is induced in the myocardium of *kl/kl* mice in relation to respective wild-type animals. **(E)** Cardiac *BNP* mRNA expression is reduced in *Hyp* mice, but upregulated in *kl/kl* mice. Values are presented as mean ± SEM; numbers in each bar graph represent the *n*-values used for the respective measurement; *P < 0.05, **P < 0.01.

In order to evaluate pro-hypertrophic genes targeted by activated calcineurin/NFAT pathway, we analyzed the expression of regulator of calcineurin 1 (Rcan1), brain natriuretic peptide (BNP), atrial natriuretic peptide (ANP), and both alpha- and beta-myosin heavy chain (aMHC, bMHC) in myocardial tissue of these two mouse models. In *kl/kl* mice, cardiac Rcan1 expression was 2.1-fold upregulated on both mRNA and protein level when compared to WT controls but not in *Hyp* mice (Figures 4A,B). The switch to a fetal cardiac gene expression pattern was only detected in *kl/kl* mice demonstrated by an enhanced *bMHC* to *aMHC* ratio when compared to respective WT littermates (Figure 4C). Moreover, the mRNA expression of the pro-hypertrophic factors *ANP* and *BNP* was clearly induced in *kl/kl* mice (5.3 ± 1.1-fold, *P* = 0.0035; 2.0 ± 0.3-fold, *P* = 0.0094) (Figures 4D,E). Interestingly, *BNP* levels were even 0.6-fold lower in *Hyp* mice in comparison to their WT controls (*P* = 0.010).

### Only klotho Hypomorphic Mice Display Enhanced Cardiac Fibrosis

**Figure 5:**
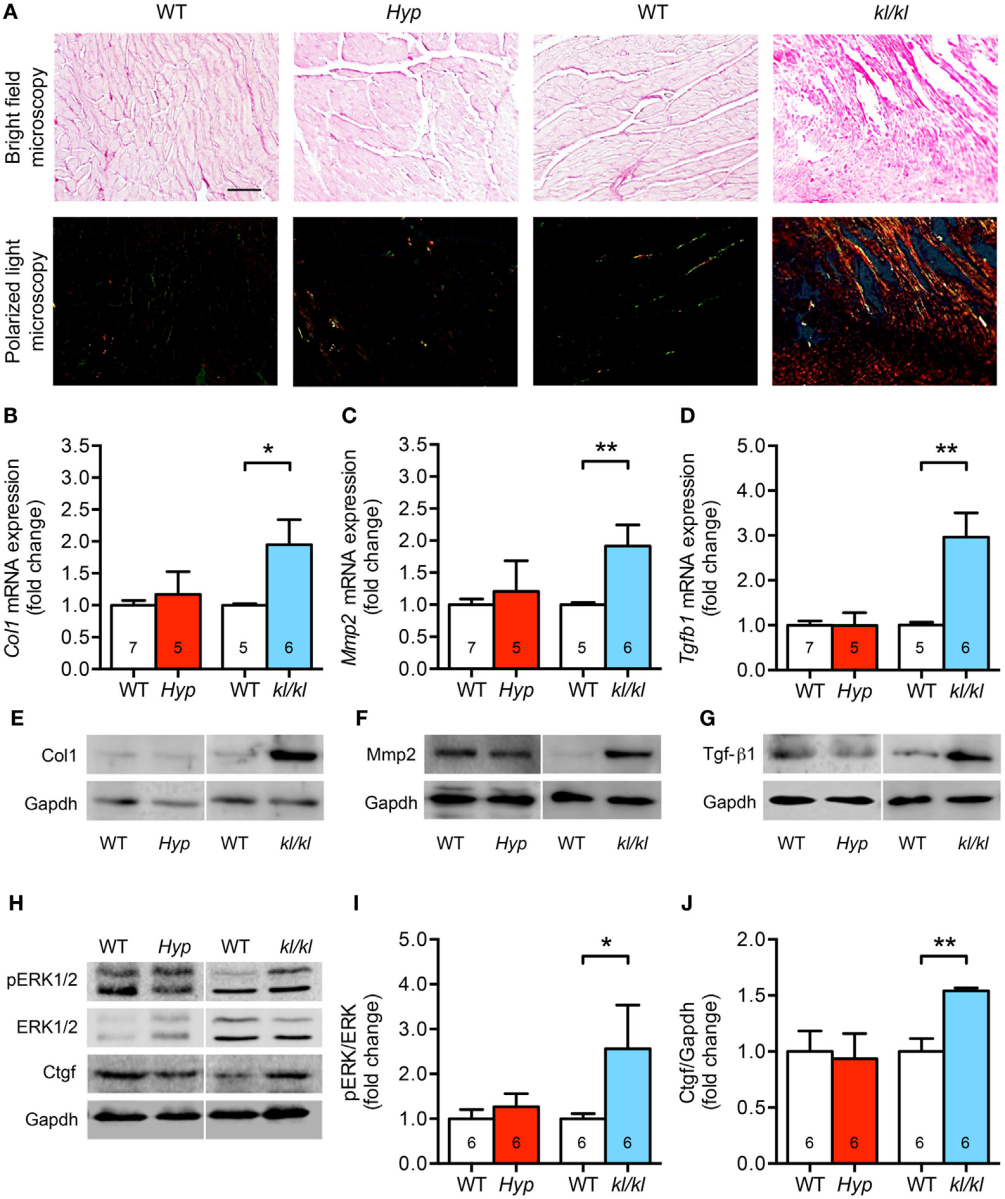
Development of cardiac fibrosis in *kl/kl* mice but not in *Hyp* mice. **(A)** Representative images of murine myocardial sections stained for fibrillar collagens by picrosirius red demonstrate increased accumulation of fibrillar collagens in *kl/kl* mice but not in *Hyp* mice (magnification, 20×; scale bar, 50 µm). **(B)** The mRNA levels of *Col1* are higher in heart tissue of *kl/kl* mice and unchanged in *Hyp* animals. **(C)** Likewise, *kl/kl* mice show an enhanced *Mmp2* mRNA expression. **(D)** The mRNA expression of *Tgfb1* is significantly different between both mouse lines whereby it is upregulated in *kl/kl* animals. The protein expression of **(E)** Col1, **(F)** Mmp2, and **(G)** Tgf-β1 in heart tissue lysates is markedly enhanced in *kl/kl* mice but not in *Hyp* mice. **(H)** ERK1/2 is only activated in *kl/kl* mice demonstrated by increased phosphorylated protein levels in immunoblot analysis followed by **(I)** densitometric quantification. **(J)** The levels of Ctgf protein are increased in *kl/kl* mice but not in *Hyp* mice. Values are presented as mean ± SEM; numbers in each bar graph represent the *n*-values used for the respective measurement; *P < 0.05, **P < 0.01.

One major characteristic of pathologic cardiac hypertrophy is the concomitant development of myocardial fibrosis (3, 41). Therefore, we next investigated collagen synthesis and remodeling as well as the expression levels of fibrosis-related molecules involved in the transforming growth factor-beta (Tgf-β) signaling cascade. Demonstrated by picrosirius red staining of myocardial tissue sections, the accumulation of fibrillar collagens was clearly increased in *kl/kl* mice but not in *Hyp* mice (Figure 5A). This was confirmed by a 2.0-fold enhanced mRNA expression of both collagen 1 (*Col1*) and matrix metallopeptidase 2 (*Mmp2*) only in *kl/kl* mice compared to WT littermates (Figures 5B,C). One major pro-fibrotic pathway in cardiomyopathy among others is the induction of connective tissue growth factor (Ctgf) *via* Tgf-β1-mediated activation of extracellular signal-regulated kinases (ERK)1/2 (41–43). Only in *kl/kl* mice, *Tgfb1* mRNA levels were 3.0-fold upregulated compared to respective WT controls (Figure 5D). The enhanced pro-fibrotic gene expressions in *kl/kl* mice were confirmed by higher Col1, Mmp2, and Tgf-β1 protein expressions in myocardial tissue of *kl/kl* mice but not in *Hyp* mice (Figures 5E–G). Moreover, ERK1/2 was activated in *kl/kl* mice demonstrated by a 2.6-fold enhanced phosphorylation in total heart tissue lysates (Figures 5H,I). Finally, Ctgf protein expression was 1.5-fold induced in *kl/kl* mice but not in *Hyp* mice (Figures 5H,J). In summary, interstitial cardiac fibrosis was only present in *kl/kl* mice.

### Mineral Metabolism Differs Between *Hyp* and *kl/kl* Mice

**Figure 6:**
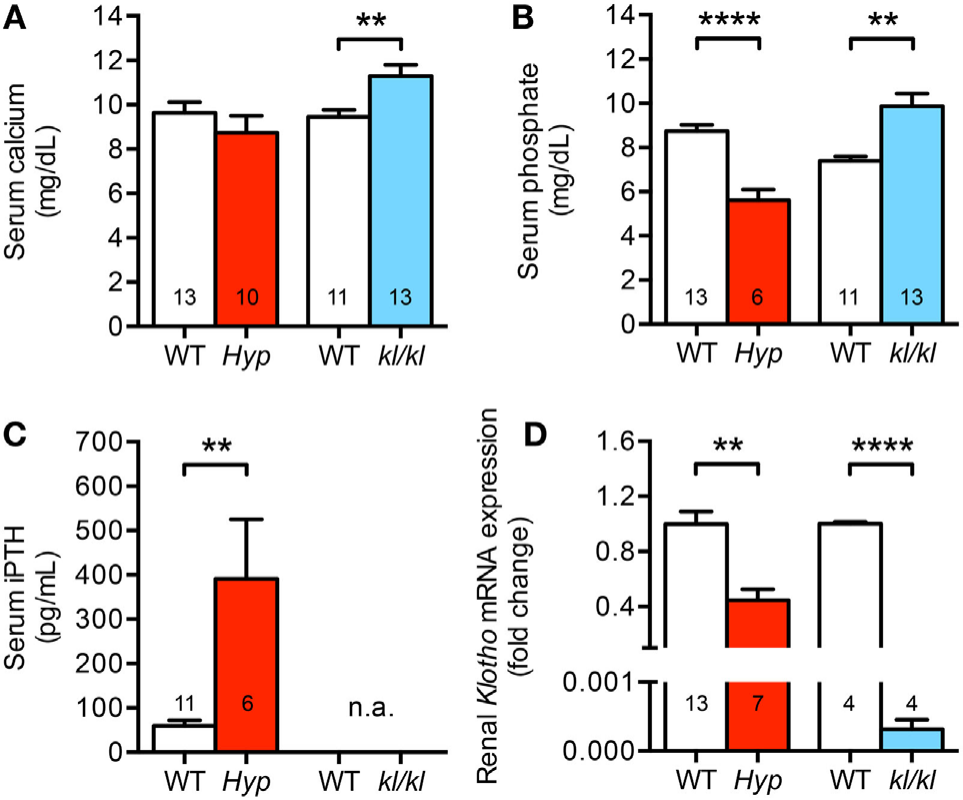
Different mineral metabolism between *Hyp* and *kl/kl* mice. **(A)** Serum calcium levels are normal to slightly reduced in *Hyp* mice and elevated in *kl/kl* animals. **(B)** *Hyp* mice are hypophosphatemic and *kl/kl* mice display hyperphosphatemia. **(C)** Serum parathyroid hormone (PTH) levels in *Hyp* mice are extensively elevated. For *kl/kl* mice, the PTH levels in the serum were not available (n.a.). **(D)** Renal *Klotho* mRNA expression is reduced in *Hyp* mice than in respective wild-type mice and barely detectable in *kl/kl* mice. Values are presented as mean ± SEM; numbers in each bar graph represent the *n-*values used for the respective measurement; **P < 0.01, ****P < 0.0001.

As we presented in this study, circulating Fgf23 levels and cardiac Fgf23 synthesis were significantly enhanced in both *Hyp* and *kl/kl* mice. However, only *kl/kl* mice developed cardiac hypertrophy and fibrosis. To identify additional causes mediating pathologic cardiac remodeling in *kl/kl* mice and to evaluate why *Hyp* mice might be protected from cardiovascular disease, we next investigated parameters of mineral metabolism. Serum calcium levels were normal to slightly reduced in *Hyp* mice (8.7 ± 0.8 versus 9.6 ± 0.5 mg/dL; *P* = 0.327) but significantly enhanced in *kl/kl* mice compared to WT littermates (11.3 ± 0.5 versus 9.5 ± 0.3 mg/dL; *P* = 0.007) (Figure 6A). In addition, *Hyp* mice displayed hypophosphatemia (5.6 ± 0.5 versus 8.7 ± 0.3 mg/dL; *P* < 0.0001) while *kl/kl* mice were hyperphosphatemic (9.9 ± 0.6 versus 7.4 ± 0.2 mg/dL; *P* = 0.0012) (Figure 6B). Furthermore, *Hyp* mice showed 6.5-fold enhanced serum PTH concentrations compared to WT animals (Figure 6C), whereas previous reports describe suppressed PTH levels in *kl/kl* mice (44, 45). Others and we showed previously that *Hyp* mice are deficient for 1,25D, whereas *kl/kl* mice have significantly higher serum 1,25D levels (37, 44–46). Finally, *Hyp* mice showed markedly reduced renal *Klotho* mRNA expression levels (0.6 ± 0.07-fold; *P* = 0.0012), which were barely detectable in renal tissue of *kl/kl* mice (Figure 6D). Taken together, despite enhanced circulating Fgf23 levels in both mouse models, serum calcium, phosphate, and 1,25D levels were increased in *kl/kl* mice. In *Hyp* mice, serum calcium was unchanged and serum phosphate and 1,25D levels were decreased compared to their WT littermates. In contrast, serum PTH levels were increased in *Hyp* mice, but suppressed in *kl/kl* mice.

### Calcium and Phosphate Stimulate Hypertrophic Cell Growth and Induce Pro-Hypertrophic Genes in Isolated Cardiac Myocytes

**Figure 7:**
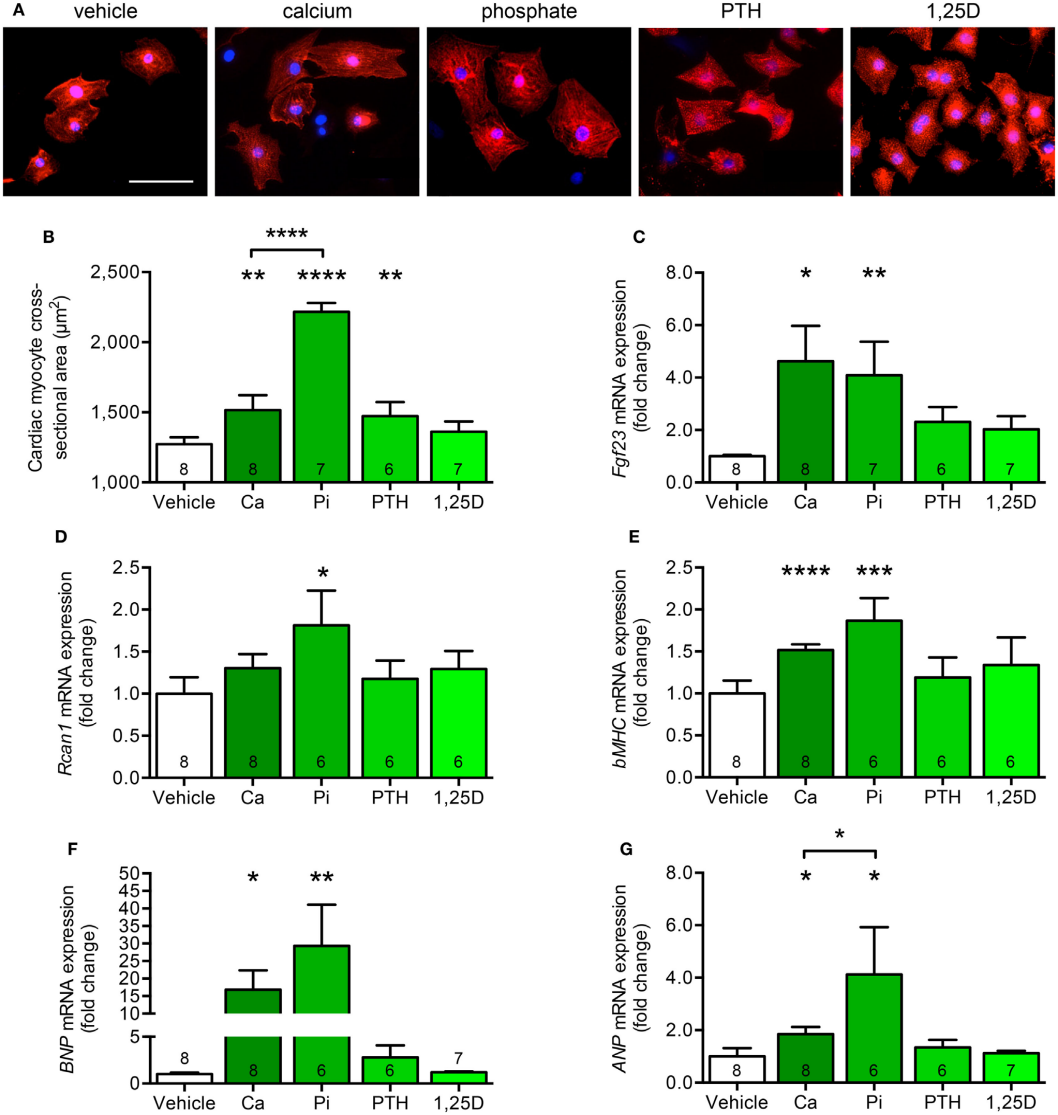
Calcium and phosphate treatment induces cell growth and pro-hypertrophic gene expression in cultured neonatal rat ventricular myocytes (NRVM). **(A,B)** Immunofluorescence staining for sarcomeric α-actinin followed by quantification demonstrates enhanced cardiac myocyte cross-sectional area after treatment of NRVM with calcium, phosphate, and PTH, but not 1,25D (magnification, 40×; scale bar, 50 µm). **(C)** Calcium and phosphate stimulation significantly increases *Fgf23* mRNA expression in NRVM. **(D)** *Rcan1* mRNA levels are significantly enhanced by treatment of NRVM with phosphate. The mRNA levels of **(E)** *bMHC*, **(F)** *BNP*, and **(G)** *ANP* are significantly upregulated after stimulation of NRVM with calcium and phosphate. Values are presented as mean ± SEM; numbers in each bar graph represent the *n-*values used for the respective measurement; *P < 0.05, **P < 0.01, ***P < 0.001, ****P < 0.0001. Abbreviations: Ca, calcium; Pi, phosphate; PTH, parathyroid hormone; 1,25D, 1,25(OH)_2_D_3_.

Since both animal models significantly differ with respect to serum calcium, phosphate, PTH, and 1,25D levels but only *kl/kl* mice developed cardiac remodeling processes (Table 2), we next investigated whether each single parameter was able to affect the endogenous *Fgf23* expression and to promote cardiac hypertrophy *in vitro*. Therefore, we stimulated NRVM, which do not express klotho, with calcium, phosphate, PTH, and 1,25D and evaluated hypertrophic growth of individual cardiac myocytes, changes of endogenous *Fgf23* expression, and finally induction of pro-hypertrophic markers. Treatment of NRVM with calcium, phosphate, and PTH but not with 1,25D significantly enhanced cardiac myocytes cross-sectional area demonstrated by immunocytochemical staining with anti-α-actinin antibody followed by quantification of the cell size (Figures 7A,B). Interestingly, *Fgf23* mRNA levels of NRVM were significantly induced by calcium and phosphate treatment but neither by PTH nor by 1,25D (Figure 7C), which is in contrast to their Fgf23-stimulating properties reported for bone (9, 47). *Rcan1* mRNA expression was significantly induced by treatment of NRVM with phosphate and tended to be increased by calcium treatment, a difference, however, not reaching statistical significance (Figure 7D). Moreover, the mRNA levels of *bMHC, BNP*, and *ANP* were significantly induced by both calcium and phosphate but not significantly modified after stimulation with PTH or 1,25D (Figures 7E–G). Taken together, among the altered parameters of mineral metabolism differing between *Hyp* and *kl/kl* mice, only calcium and phosphate clearly induced cardiac *Fgf23* expression and promoted myocytes hypertrophy *in vitro*, suggesting that elevated phosphate and calcium levels may promote Fgf23-mediated cardiac hypertrophy in states of klotho deficiency.

**Table 2:** Differences between *Hyp* and *kl/kl* mice regarding characteristics of cardiovascular and kidney disease, cardiac FGF23/FGFR4 system, and parameters of mineral metabolism.

Characteristics	*Hyp* mouse	*kl/kl* mouse
Cardiac hypertrophy	–	+
Cardiac fibrosis	–	+
Kidney injury (48, 49)	–	–
Blood pressure (44, 50)	↑	↓
Calcification (44, 51)	–	+
Serum Fgf23	↑	↑↑
Cardiac Fgf23	↑	↑
Cardiac Fgfr4	↔	↑
Renal klotho	↓	↓↓
Serum calcium	↔ ↓	↑
Serum phosphate	↓	↑
Serum parathyroid hormone (44, 51)	↑	↓
Serum 1,25D (37, 44, 51)	↓	↑↑

## Discussion

High FGF23 levels and klotho deficiency are postulated as key risk factors for the development of uremic cardiomyopathy (2, 23, 25, 31, 52) and increase of FGF23 is further associated with poor outcome in patients with heart failure, cardiogenic shock, or arrhythmia at normal kidney function (4–8). Even though FGF23 directly targets the heart and contributes to hypertrophy and fibrosis independent of klotho (27, 28), it is not known whether the increase of FGF23 in parallel with reduction of klotho alone is sufficient to lead to CVD. Here, we show that enhanced FGF23 synthesis in *kl/kl* mice led to the development of cardiac hypertrophy and fibrosis while *Hyp* mice, despite of high circulating and cardiac FGF23 levels and reduced renal klotho expression, did not show any cardiac phenotype. This suggests that elevated FGF23 levels in the presence of klotho deficiency do not necessarily promote cardiac toxicity of FGF23 *in vivo per se*. Among additional key factors of altered mineral metabolism in *Hyp* and *kl/kl* mice, only calcium and phosphate stimulated endogenous FGF23 expression in cultured cardiac myocytes and induced pro-hypertrophic gene expression resulting in hypertrophic cell growth *in vitro*. The present results suggest that the differences in serum calcium and phosphate levels between the two mouse models may contribute to the effects on cardiac hypertrophy and fibrosis at high FGF23 levels. Concomitant normal serum calcium levels and hypophosphatemia may prevent *Hyp* mice from the development of LVH, whereas concomitant hypercalcemia and hyperphosphatemia may have further promoted pathological cardiac remodeling in *kl/kl* mice.

In the present study, both *Hyp* and *kl/kl* mice presented with enhanced circulating levels of C-term and intact FGF23 as well as upregulated cardiac FGF23 synthesis in addition to reduced renal *Klotho* expression, although both alterations were more pronounced in *kl/kl* mice. However, *kl/kl* mice, but not *Hyp* mice, developed cardiac hypertrophy, demonstrated by an increase in relative heart weight and cardiac myocyte cross-sectional area. In addition, we demonstrated for the first time that FGFR4 is upregulated in heart tissue of *kl/kl* mice and this resulted in activation of calcineurin/NFAT signaling and finally induction of pro-hypertrophic NFAT target genes *Rcan1, bMHC, ANP*, and *BNP*. Furthermore, *kl/kl* mice further displayed enhanced myocardial fibrosis with concomitant induction of collagen 1, Mmp2 and Tgf-β1 expression, activated ERK1/2 and Ctgf protein. Thereby, we support the findings of Hu et al. showing that pERK1/2 is elevated and myocardial fibrosis is present in heart tissue of homozygous *kl/kl* mice as well (31). Moreover, this group investigated the Tgf-β1-mediated activation of ERK1/2 in neonatal rat cardiac myocytes and fibroblasts *in vitro*, which was only present in the absence of klotho suggesting that there is a strong connection between klotho deficiency and the induction of pro-fibrotic pathways. In addition, treatment of neonatal mouse cardiac fibroblasts with the 65 kDa soluble klotho isoform was shown to suppress myofibroblast proliferation and collagen synthesis (33). Taken together, our data suggest that the well-established cardiac FGF23/FGFR4 signaling involved in the development of pathologic cardiac hypertrophy with the parallel appearance of cardiac fibrosis is induced in *kl/kl* mice with high FGF23 levels and klotho deficiency.

Our results in homozygous *kl/kl* mice are well in line with Yang and colleagues who showed that heterozygous *kl/*+ mice had elevated relative heart weight and enhanced left ventricular posterior wall thickness with reduced left ventricular internal diastolic diameter (35). In another study by Hu et al., heterozygous *kl/*+ mice showed reduced ejection fraction, stroke volume, and cardiac output in addition to enlarged septum and posterior wall thickness within the diastole (31). In contrast, neither *kl/*+ nor *kl/kl* mice with comparable age had baseline cardiac abnormalities published in other studies by Xie and colleagues (32, 53). Interestingly, according to this study, serum FGF23 and phosphate levels were similar in WT and *kl/*+ mice. The different cardiac outcome in klotho-deficient mice within these studies might be, at least partially, due to differences in dietary phosphate content ranging from 0.35 to 0.6% (31, 32, 35, 53).

The development of pathologic cardiac remodeling including hypertrophy and fibrosis is less clear in *Hyp* mice. Recently, it was described that 8-week-old *Hyp* mice showed increased relative heart weight, and administration of an FGFR1 activating antibody resulted in normalization of relative heart weight, left ventricular wall thickness, and blood pressure. Treatment of *Hyp* mice with soluble klotho ameliorated systolic, diastolic, and mean arterial blood pressure (MAP) (54). Andrukhova and colleagues showed enhanced FGF23 serum levels in 3-month-old *Hyp* mice with enhanced MAP in addition to significantly higher relative heart weight compared to WT littermates (50). Thus, both studies suggest that *Hyp* mice on a regular phosphate diet present with enhanced blood pressure and cardiac hypertrophy. However, a detailed evaluation of the cardiac phenotype in *Hyp* mice, i.e., investigation of cardiac myocyte cross-sectional area, pro-hypertrophic signaling pathways, and concomitant fibrosis, in order to evalute pathological cardiac hypertrophy in-depth, was not yet been performed in *Hyp* mice. In the present study, we investigated histological and molecular biological analyses of heart tissue in 6- to 8-week-old *Hyp* mice on a regular phosphate diet and compared our findings to their WT littermates. In contrast to the abovementioned studies (50, 54), relative heart weight was not significantly altered in *Hyp* mice in the present study, and additionally, cardiac myocyte cross-sectional area was similar to controls. Although, *Hyp* mice showed comparably increased circulating FGF23 levels and the presence of enhanced cardiac FGF23 synthesis in the present study, FGFR4 and its respective downstream signaling pathway, i.e., calcineurin/NFAT, *Rcan1, bMHC, ANP*, and *BNP*, were not induced at all. Neither accumulation of fibrillar collagens nor fibrosis-related pathways, including collagen 1 expression, Tgf-β1-mediated activation of ERK1/2, or Ctgf were induced in *Hyp* mice compared to controls. Thus, our data point out that high FGF23 levels in addition to klotho deficiency does not necessarily result in pathological cardiac remodeling *in vivo*. The fact that blood pressure is elevated in *Hyp* mice (50) while low in *kl/kl* mice (44) further supported the hypothesis that FGF23-mediated cardiac hypertrophy might be blood pressure independent.

Therefore, the question arises if alterations in the mineral metabolism may modulate the cardiac phenotype in these two mouse models. In line with previous published studies by others (37, 55) and us (46), *Hyp* mice showed normal to reduced serum calcium levels, presented with hypophosphatemia, secondary hyperparathyroidism, and vitamin D deficiency. In contrast, *kl/kl* mice were hypercalcemic and hyperphosphatemic, had elevated 1,25D levels, and suppressed serum PTH levels (44, 45). Thus, the observed differences in calcium and phosphate serum concentrations may at least partly explain the different cardiac phenotypes in the two mouse models. The importance of phosphate as a risk factor for cardiomyopathy was previously demonstrated in a variety of experimental studies showing that high-phosphate diet induced cardiac hypertrophy and fibrosis in rodents (25, 28, 56). Moreover, stimulation with phosphate induced pERK1/2, Ctgf, and collagen 1 in isolated neonatal rat cardiac fibroblasts and in addition pERK1/2, Ctgf, and pSmad2/3 in cardiac myocytes *in vitro* (31). Interestingly, co-treatment with soluble klotho only ameliorated Ctgf and collagen 1 levels in cardiac fibroblasts and Smad2/3 phosphorylation in myocytes suggesting that klotho primarily targets phosphate-induced pro-fibrotic signaling pathways. The impact of phosphate on the development of cardiac hypertrophy on cellular level was not investigated so far. Here, we present that both calcium and phosphate stimulated hypertrophic growth of cultured cardiac myocytes with upregulation of *Rcan1, bMHC, ANP*, and *BNP*, respectively. Interestingly, among the known parameters of mineral metabolism that induce FGF23 synthesis in bone (9), only calcium and phosphate upregulated endogenous *Fgf23* expression in NRVM.

Circulating FGF23 levels were considerably higher and the degree of klotho deficiency was lower in *kl/kl* mice compared to *Hyp* mice in the present study. Therefore, we cannot exclude that further stimulation of circulating FGF23, e.g., by increased phosphate load may result in pathological cardiac remodeling in *Hyp* mice despite the presence of hypophosphatemia. In addition, *kl/kl* mice presented with hypercalcemia, which was shown to stimulate cardiac myocyte hypertrophy *in vitro*, although the effects of calcium were lower compared to phosphate. However, concomitant hypercalcemia may have further promoted the cardiac phenotype in *kl/kl* mice. Furthermore, in *kl/kl* mice, the regulation of cardiac hypertrophy and fibrosis by other pathological factors cannot be ruled out (57–59). Clearly, further studies are needed to confirm an association of altered mineral metabolism at high FGF23 levels and pathological cardiac remodeling.

In extension to previously published studies, our results support the hypothesis that both altered parameters of mineral metabolism and elevated FGF23 levels contribute to cardiac hypertrophy and fibrosis in the setting of klotho deficiency, and consequently, *Hyp* mice might be protected from pathologic cardiac remodeling by the presence of concomitant hypophosphatemia and lack of hypercalcemia. In addition, high FGF23 levels are only cardiotoxic in the presence of high calcium and/or high phosphate and independent of klotho deficiency. The latter supports the concept of early initiation of phosphate lowering treatment in states of elevated FGF23 and klotho deficiency, e.g., uremia, in order to prevent pathological cardiac remodeling.

## Ethics Statement

All experimental procedures were approved by the State Office committee on animal welfare Lower Saxony for *Hyp* mice and Baden-Württemberg for *kl/kl* mice, and performed in accordance with national animal protection guidelines from Directive 2010/63/EU of the European Parliament on the protection of animals used for scientific purposes.

## Author Contributions

Design: ML-N, BR, JV, and DH; experiments: BR, MB, JN, and IV; experimental support and critical revision of the manuscript: IA, JV, FL, JH, and SK; data evaluation: ML-N, BR, MB, JN, and IV; preparation of manuscript: ML-N, BR, and DH.

## Conflict of Interest Statement

The authors declare that the research was conducted in the absence of any commercial or financial relationships that could be construed as a potential conflict of interest.
